# Pregnancy and Prenatal Management of Congenital Adrenal Hyperplasia

**DOI:** 10.3390/jcm11206156

**Published:** 2022-10-19

**Authors:** Gianluca Cera, Pietro Locantore, Roberto Novizio, Ettore Maggio, Vittoria Ramunno, Andrea Corsello, Caterina Policola, Paola Concolino, Rosa Maria Paragliola, Alfredo Pontecorvi

**Affiliations:** 1 Unit of Endocrinology, Department of Translational Medicine and Surgery, Università Cattolica del Sacro Cuore—Fondazione Policlinico “A. Gemelli” IRCCS, Largo Gemelli 8, 00168 Rome, Italy; 2 Unit of Clinical Chemistry, Biochemistry and Molecular Biology, Department of Laboratory and Infectiology Sciences, Università Cattolica del Sacro Cuore—Fondazione Policlinico “A. Gemelli” IRCCS, Largo Gemelli 8, 00168 Rome, Italy; 3 Unicamillus, Saint Camillus International University of Medical Sciences, Via di S. Alessandro 10, 00131 Rome, Italy

**Keywords:** congenital adrenal hyperplasia, pregnancy, prenatal therapy, genetic testing, hydrocortisone, dexamethasone, prednisone

## Abstract

Congenital adrenal hyperplasia (CAH) is a group of autosomal recessive diseases that may cause cortisol insufficiency together with other hormonal alterations. The most common form is 21-hydroxylase deficiency, in which the lack of pituitary negative feedback causes an increase in ACTH and adrenal androgens. Classical forms of CAHs can lead to severe adrenal failure and female virilization. To date, the appropriate management of pregnant CAH patients is still debated regarding appropriate maternal therapy modifications during pregnancy and the risks and benefits of prenatal treatment of the fetus. We conducted a literature search of relevant papers to collect current evidence and experiences on the topic. The most recent and significant articles were selected, and current international guidelines were consulted to update current recommendations and guide clinical practice. Given the lack of randomized clinical trials and other high-quality scientific evidence, the issue is still debated, and great heterogeneity exists in current practice in terms of risk/benefit evaluation and pharmacological choices for pregnancy and prenatal treatment. Glucocorticoid therapy is advised not only in classical CAH patients but also in non-classical, milder forms. The choice of which glucocorticoid to use, and the safety and benefits of dexamethasone therapy aimed at preventing genital virilization are still debated issues. Several advances, however, have been made, especially in terms of fertility and reproduction. This review aims to present the most recent scientific and real-world updates on pregnancy and prenatal management of CAH, with the presentation of various clinical scenarios and specific case-by-case recommendations.

## 1. Congenital Adrenal Hyperplasia

Congenital adrenal hyperplasia (CAH) is a group of autosomal recessive endocrine disorders characterized by a defect in one or more steps of adrenal steroidogenesis, with subsequent defective synthesis of cortisol, ACTH excess, accumulation of precursors, and their shunting through alternative pathways. Chronic ACTH excess leads to adrenal enlargement. The clinical features of these patients depend on (1) the severity of cortisol deficiency, (2) the presence and severity of other hormonal deficits, and (3) the hormonal excess resulting from the hyperactivation of the remaining adrenal functioning pathways. A genotype–phenotype correlation exists for many but not all of the described mutations, with some genotypes showing variable clinical severity, possibly because of an interplay with different genetic backgrounds [1,2].

**Figure 1:**
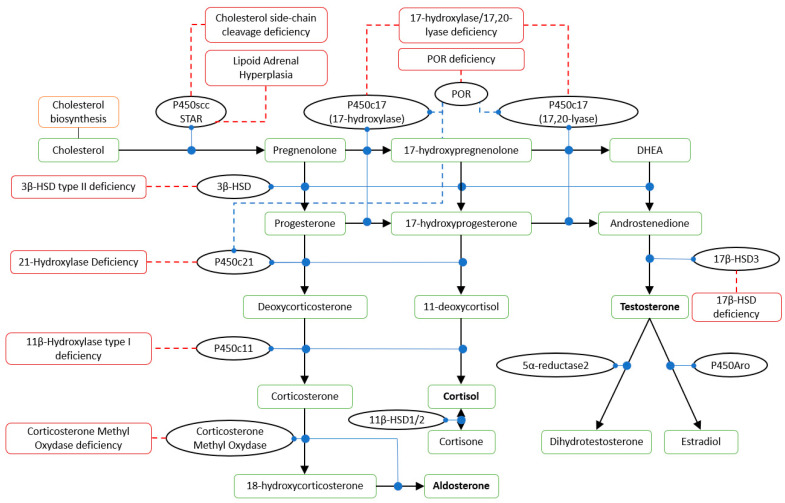
Steroid hormones synthesis pathway. P450scc: cholesterol side-chain cleavage enzyme; STAR: steroidogenic acute regulatory protein; POR: cytochrome P450 reductase; P450c17: steroid 17 alpha-hydroxylase/17,20 lyase; HSD: hydroxysteroid dehydrogenase; P450c21: 21-hydroxylase; P450c11: 11β-hydroxylase; P450Aro: aromatase. Solid black arrows: conversion; solid blue circles: catalysis; dashed blue lines: stimulation; solid dashed red lines: associated disorders.

21-hydroxylase deficiency (21-OHD) is the most common form of CAH, accounting for 95–99% of cases [1,3]. This form is caused by *CYP21A2* variants that impair the synthesis of 11-deoxycortisol and deoxycorticosterone (DOC), with a resulting lack of cortisol and aldosterone secretion and the accumulation of 17-OH-progesterone (17-OH-P) and progesterone, respectively (Figure 1), producing hyperandrogenism.

Classical 21-OHD has an incidence of 1:10,000–15,000 live births and is caused by homozygous or compound heterozygous mutations, provoking severe cortisol deficiency. Around 75% of patients show a severe mineralocorticoid deficiency too, and present with classical “salt-wasting” forms, which can translate in perinatal-onset adrenal crisis. The remaining 25% of patients, who have sufficient aldosterone activity, present with classical “simple-virilizing” 21-OHD. These two forms should be regarded as the extremities of a continuum of phenotypes rather than two separate categories [1]. Genital virilization can be found in female newborns affected by both forms of classical 21-OHD because of high androgen levels during sexual development stages (9th–15th gestational week).

Non-classical 21-OHD usually presents in a milder form. It has a prevalence of 1:300 to 1:27 in different ethnic groups, potentially making 21-OHD one of the most common autosomal recessive diseases in humans [4]. This form arises from partial loss-of-function mutations, with reduced but valid enzymatic activity, and a clinical picture mainly arising from hyperandrogenism. Indeed, these patients show no risk of adrenal crisis if not on glucocorticoid treatment [5] nor genital abnormalities at birth.

Hyperandrogenism of both classical and non-classical 21-OHD can result in hyperandrogenic cutaneous manifestations such as acne and hirsutism, frontal/temporal alopecia, hypostaturalism, delayed menarche, and central precocious puberty, and possibly a certain grade of masculinization of the body and the central nervous system [1,6,7,8]. Both sexes can present impaired fertility because of menstrual irregularities, secondary PCOS, anovulation, or genital abnormalities in females and because of reduced sperm counts, low testosterone (resulting from androstenedione suppressing gonadotropins), and testicular adrenal rest tumors (TARTs) in males [9]. TARTs are benign, mostly bilateral lesions that arise in 30–86% of males affected by classical CAH but have also been described in non-classical CAH and in other conditions characterized by ACTH excess, such as Addison disease, ACTH-dependent Cushing’s syndrome, and Nelson’s syndrome [10,11,12]. If untreated, TARTs can grow and impair testicular function because of gonadotropin suppression and/or seminiferous tubule obstruction [13].

The severity of all these symptoms depends on the entity of the endocrine alterations in each patient, and they may be attenuated or absent when the patient is adequately treated [1,4,14].

The diagnosis of classical 21-OHD is usually obtained at birth on the basis of clinical signs and symptoms. The confirmation test is based on neonatal screening of 17-OH-P levels and, if needed, a corticotropin stimulation test. Cortisol and renin-angiotensin-aldosterone must be evaluated. Non-classical 21-OHD can be diagnosed at any age but most frequently after puberty. The diagnosis is based on the basal and/or stimulated levels of 17-OH-P and needs to be measured in the early follicular phase (third to fifth day) of the ovarian cycle if the patient menstruates [15,16]. Following biochemical diagnosis, patients may undergo genetic testing for *CYP21A* (Figure 1) [10]. Healthy patients carrying heterozygous mutations may show no hormonal alterations and can be diagnosed via genotyping only [2]. The genetics of CAH has been extensively reviewed by Hannah-Schmouni et al. and more recently revised by Narasimhan and Khattab [2,17].

Other forms of enzymatic deficits are much rarer than 21-OHD; their typical features are outlined in Table 1 [1,18,19]. Rarely, “non-classical”, late-onset cases of these other forms of CAH have been described, usually caused by incomplete loss-of-function mutations with some residual enzymatic activity [1,20].

Treatment of CAH must be personalized for each patient because of the broad variability of clinical features.

Sex assignment in the case of virilized female genitalia (or undeveloped genitalia in rare CAH forms) must be evaluated comprehensively, considering the patient’s genotype, endocrine alterations, potential for future sexual activity and fertility, and potential mental health consequences [1]. Sex assignment should be held by a skilled team to avoid misdiagnosis.

In non-classical 21-OHD, glucocorticoid therapy may be used in more severe cases or in patients seeking a pregnancy to lower ACTH and androgen levels [5]. In classical CAH, instead, exogenous glucocorticoids are permanently required. As for other forms of adrenal insufficiency, patients and their caregivers must be educated on stress therapy, emergency treatment, and appropriate diet and hydration [1]. For pediatric patients, hydrocortisone at 10–15 mg/m^2^/day fractioned in 3–4 doses is the recommended glucocorticoid [1,16]. Such dosage may be slightly increased during puberty. Adult patients usually require hydrocortisone at 15–25 mg/day fractioned in 2–3 doses. In Addison’s disease, dual-release hydrocortisone has demonstrated an improved metabolic profile and quality of life [21], especially when administered in fractionated daily doses [22]. Similar hydrocortisone modified-release formulations have been developed for CAH patients, showing an efficient suppression of the early morning ACTH peak and a more physiological circadian rhythm [23]. Anecdotal data support the feasibility of continue subcutaneous hydrocortisone infusion [24], even though it is yet to be foreseeable in real-world practice [1]. Other than hydrocortisone, prednisone, prednisolone, and methylprednisolone twice daily and dexamethasone once daily are being used. 21-OHD patients show adequate androgen suppression when treated with dexamethasone [21,25]. However, dexamethasone and, to a lesser extent, prednisone, are associated with worse long-term outcomes compared to hydrocortisone in classical 21-OHD [26]. Moreover, dexamethasone is not inactivated by placental enzymes, leading to fetal exposure to glucocorticoids (see Section 3.4.1). In CAH patients with evidence of mineralocorticoid deficiency, fludrocortisone (0.05–0.2 mg daily) is added, with appropriate dietary salt supplementation. The use of GH, GnRH analogues, aromatase inhibitors, estrogen-progestins, and/or anti-androgens has been reported in CAH [1,10,12]. New formulations and innovative genetic therapies are being studied, with promising results [17].

Hormonal therapy must be carefully titrated and monitored, with regular clinical and laboratory follow-up [1,16]. An increase in glucocorticoid replacement therapy may be advisable in male patients affected by CAH presenting with growing TARTs to reduce ACTH levels, weighing such benefit against the risks of glucocorticoid excess. Cryopreservation should be considered; surgery may be contemplated for larger TARTs, although there is no clear evidence of beneficial outcomes [9].

Even if most CAH patients have reduced fertility, pregnancy is possible, but it requires adequate management to avoid irreversible harm to the pregnant patient and delivery, to the embryonal and fetal development, and at birth. Successful pregnancies have been described in most but not all subtypes of CAH, sometimes only upon hormonal stimulation and/or other assisted reproductive technology [6].

This introduction briefly presented the challenges of CAH management. In pregnancy, several new factors come into play: the fetal risk of inheriting CAH mutations; the different impact of such mutations based on the genetic sex of the fetus; the risk of fetal adrenal insufficiency and sexual abnormalities based on the excess or lack of treatment; the benefit/risk ratio of starting or modifying a glucocorticoid therapy for both the patient and the fetus; and the ethical concerns in exploring different treatment strategies. Current international guidelines underline that further research is needed on prenatal treatment, how glucocorticoid requirements change during pregnancy, and the risk/benefit ratio of glucocorticoid therapy for non-classical 21-OHD patients [16]. Such issues further complicate clinical and therapeutic decisions. Here, we present a review of the current literature to gather information and guidance for clinicians facing these challenges.

## 2. Methods

A literature search was conducted via Pubmed.gov to find relevant papers related to the management of pregnancy in CAH patients, including the following keywords: congenital adrenal hyperplasia, pregnancy, prenatal therapy, genetic testing, hydrocortisone, dexamethasone, prednisone. National and international guidelines for the management of CAH and Addison’s disease (when pertinent) were also consulted and reviewed.

## 3. CAH and Pregnancy

It is necessary to point out that, due to ethical concerns, no clinical trial has been or is being carried out, to date, to evaluate the different treatment strategies available in the management of CAH during pregnancy, so there is no high-quality scientific evidence to issue specific recommendations for the following scenarios. Expert opinions based on pathophysiological and pharmacological considerations diverge on some recommendations (see below).

### 3.1. Fertility and CAH

A retrospective study based on an American population showed potentially higher infertility rates, lower rates of successful pregnancies, and higher risks of caesarean section, chorioamnionitis, maternal infections, and small-for-gestational-age newborns in patients with CAH [27]. This study, however, assumed a CAH diagnosis based on the presence of an ICD-9 code 255.2, with no possibility of distinguishing the various forms of CAH. In fact, other studies showed different results (see below). In any case, current data indicate that appropriate clinical management can mitigate and even cancel these risks [6].

Regarding 21-OHD, a large Swedish cohort study, including patients with confirmed 21-OHD diagnoses, showed a reduced birth rate (especially in salt-wasting forms), a lower rate of children per woman, and a slightly older age at first delivery; moreover, the authors observed an increased risk of gestational diabetes, possibly because of glucocorticoid treatment, and of cesarean section, especially in salt-wasting forms. This study, however, showed similar perinatal outcomes between CAH pregnancies and controls, and no increased rates of small-for-gestational-age newborns, except for simple virilizing forms compared to non-classical forms [28]. The observed reduced birth rate may be explained by a reduced interest in motherhood and other social-behavioral aspects, such as the lack of a relationship and/or a higher proportion of homosexuality. In fact, in classical 21-OHD, fertility rates among patients who do wish to conceive are comparable to the general population [19]. Other factors that may come into play are an unfavorable congenital/post-surgical anatomy with subsequent difficulties at vaginal intercourse and/or lack of appropriate information and education (almost half of CAH patients may have inadequate awareness on their fertility and their chances to conceive). Moreover, inadequate glucocorticoid treatment may lead to androgens and progesterone excess, with subsequent disruption of GnRH pulses and LH pulses, altered ovarian androgen secretion (i.e., PCOS-like phenotype) and/or cervical mucus thickening, amenorrhea, and anovulation [9,19]. In fact, a slight overtreatment of patients with classical CAH may increase the chances of a pregnancy [6]. Some authors have reported the use of fludrocortisone even in simple-virilizing classical 21-OHD patients, with a reduction in 17-OH-P levels, possibly because of an underlying subclinical mineralocorticoid deficiency [29]. As for non-classical 21-OHD, fertility can be compromised by oligo-ovulation, mainly because of altered GnRH pulses and subsequent decreased LH levels; usually, low-dose glucocorticoids are sufficient to control hyperandrogenism and facilitate pregnancy in these cases [30].

Successful pregnancies have been reported in appropriately treated 11-OHD patients, possibly after hormonal stimulation [6,31,32,33].

As for type 2 3BHSD deficiency, no pregnancy and fertility rates have been published to date [34].

Patients affected by POR deficiency present with inconsistent phenotypes because of impaired adrenal and gonadal enzyme activities, with residual synthesis of sex steroids from the accumulated precursors by other enzymes (backdoor pathway) [35], with a variable grade of external genitalia development (that can be normal) with mild female virilization and/or male hypovirilization, and low levels of postnatal androgens. Four pregnancies obtained via assisted reproductive technology have been reported to date [36,37,38].

The remaining CAH forms (17OH/17,20-lyase, StAR, and P450scc deficiencies) are not associated with hyperandrogenism and virilization; conversely, they show reduced androgens with subsequent incomplete or absent sexual development. 46,XX 17-OHD patients have reduced fertility rates because of reduced estrogens and increased progesterone, with anovulatory cycles, primary amenorrhea, a high incidence of ovarian cysts, and a reduced uterine volume. Pregnancies have been described in patients treated with dexamethasone, GnRH analogues, and/or hCG and hMG, to reduce progesterone levels, prior to ovarian stimulation [39,40,41,42]. Female patients affected by StAR deficiency show normal pubertal development because of normal ovarian hormone secretion, but ovulation and embryo implant may be impaired, causing infertility. Three successful pregnancies after ART have been reported to date [43,44,45]. Moreover, “nonclassical”, less severe lipoid adrenal hyperplasia phenotypes have been described, with both natural and stimulated successful pregnancies reported [46]. We found no reports of pregnancies in P450scc deficiency patients.

### 3.2. Preconception Education and Genetic Counselling

Most authors underline the importance of patient education and counselling [1,6,16,47,48]. Patients with CAH should be made aware of the consequences of CAH on fertility, reproduction, and pregnancy as presented above. Nonetheless, they should be reassured that current data indicate that appropriate clinical management can lower and even cancel these risks, especially for 21-OHD patients [6]. Moreover, appropriate psychosexual counselling and advanced genital surgery techniques may result in improved fertility rates [19].

Preconception genetic counselling is mandatory for CAH patients to inform them on the risk of passing CAH onto their offspring. Being an autosomal recessive disease, CAH has a mendelian risk of vertical parents to fetus transmission that depends on the genotype of the biological parents of the fetus. If both parents carry a mutation, newborns are at risk of classical CAH; therefore, the fetus may be tested for karyotype and *CYP21A2* analysis (via chorionic villus sampling, amniocentesis, or cell-free fetal DNA testing, see below) to evaluate the subsequent risk of (1) adrenal insufficiency and (2) adrenal crisis, (3) hyperandrogenism, and subsequent (4) genital development abnormalities and (5) consideration of prenatal therapy. Patients with CAH should be informed on the possible need of testing partners before conception and planning pregnancies, presenting all these risks and their consequences. In fact, unexpected pregnancies may impede appropriate genetic testing of the partner and fetal risk evaluation. Planned pregnancies, instead, allow for genetic counselling and timely testing of the partner, which is recommended for couples desiring to have children [16,49]. The appropriate partner testing and the prompt identification of a pregnancy allow planning for genetic tests on the fetus too. Moreover, appropriate therapy modifications may be indicated (e.g., switching from dexamethasone to other corticosteroids; see Section 3.4.1) and planned pregnancies avoid the risk of inappropriate fetal exposure to glucocorticoids.

### 3.3. Fetal Genetic Testing

Fetal genetic testing is usually performed around the 10th to 13th gestational week via chorionic villus sampling, but it can also be performed with amniocentesis at later times (15th to 16th gestational week) [16,50]. Given that genitalia formation begins around the ninth gestational week [51], starting a prenatal therapy after such time may be too late to prevent development abnormalities, posing clinicians and patients the dilemma of whether to undergo prenatal therapy, with a variable but usually substantial risk of inappropriate exposure of non-affected fetuses, or to refuse it, with subsequent risk of virilized female fetuses and related consequences.

The collection of fetal DNA samples in maternal peripheral blood may be used as early as the sixth gestational week to identify the presence of Y-chromosomes to exclude male fetuses from prenatal therapy (sex typing) [51,52]. Massively parallel sequencing of cell-free fetal DNA may also allow *CYP21A2* genotyping, leading to an earlier diagnosis, so that non-biallelic-mutated and male fetuses may be excluded from (or less exposed to) prenatal therapy [49,51]. Such techniques, however, are still expensive and often unavailable: Nowotny et al. reported the use of early sex typing in 11/13 tertiary care European centers and *CYP21A2* genotyping in only 1/13 of these centers [50]. Nonetheless, it is foreseeable that the costs and availability of these diagnostic tests may improve over time, expanding their use, and thus improving the benefit/risk ratio of such therapy [53,54].

### 3.4. Pharmacologic Treatment during Pregnancy

#### 3.4.1. General Considerations and Recommendations

Hydrocortisone, prednisone, and prednisolone are inactivated by the placental type 2 11β-HSD; dexamethasone is not inactivated and should therefore be used in pregnant patients only if a fetal effect is required (i.e., in the case a prenatal therapy has been chosen, see below); in all other cases, dexamethasone should be discontinued and switching to hydrocortisone or other corticosteroids is recommended, with appropriate equivalent dosage, without modifications of the usual maintenance dose during the first two trimesters. In patients with adrenal insufficiency, glucocorticoid replacement therapy is usually increased by 20–40% during the third trimester; similar recommendations exist for CAH [48]. It is of note that 17-OH-P tends to physiologically increase throughout the pregnancy, whereas androstenedione increases, reaching a plateau at the 12th gestational week. Dose titration therefore requires trimester-appropriate reference ranges. The daily schedule of glucocorticoid administration is still debated. A “reverse circadian rhythm” administration, with a larger dose in the evening, could in theory obtain a better reduction in ACTH and androgen activity, though losing the possibility of mimicking the physiological circadian rhythm of cortisol production. However, a clear benefit of one timing schedule versus the other has not yet been observed [55,56].

Mineralocorticoid requirements tend to increase during pregnancy too, given the anti-mineralocorticoid effects of the increased progesterone levels, and blood pressure and serum potassium should be used to titrate fludrocortisone doses instead of renin, which is unreliable during pregnancy [6,57,58].

As for other stressing events, labor and delivery require an increase in glucocorticoid administration. Current recommendations consist of 100 mg i.m./i.v. hydrocortisone at the onset of active labor, followed by 200 mg/24 h in fractioned doses (both orally or iv). The dose may be increased in case of complications [6,48]. After delivery, an orally administrated double dose should be maintained for 2–4 days [48]; hydrocortisone should usually be reduced to 100 mg/24 h in four daily doses during the first day post-partum and 50 mg in three daily doses during the second day post-partum. No specific protocols exist for following further reduction, but pre-pregnancy doses can be restored if there are no clinical complications [6,48].

Breastfeeding is recommended [48], with some authors suggesting breastfeeding before taking hydrocortisone to reduce the already minimal concentration excreted in breast milk [6].

#### 3.4.2. Prenatal Therapy

Prenatal therapy regimens, since their first proposal in 1984 [59], have not been validated by solid studies. If prenatal treatment is chosen, as presented below, it is recommended that it is carried out in the context of experimental therapies in referral centers, with appropriate long-term follow-up registries, including prenatally treated children and adults [16]. Based on embryology considerations, such therapy should be started by the ninth gestational week to be effective in avoiding hyperandrogenism and subsequent genital development abnormalities in female fetuses. Dexamethasone is usually administered for this prenatal treatment at the dosage of 20 μg/kg/day (based on pre-pregnancy body weight), fractioned in one, two, or usually three daily doses, up to 1.5 mg/day [50,60]. Recently, Stachanow et al. proposed a markedly reduced dose of 7.5 μg/kg/day [61]. Such therapy should be started by the 6–8th and continued up to the 16th gestational week minimum in order to be effective.

The benefit/risk ratio of prenatal glucocorticoid treatment is still controversial because of safety concerns [1].

The expected benefits of prenatal therapy depend on the suppression of fetal ACTH, which avoids fetal hyperandrogenism and female genital virilization in at least 80–85% of cases [62]. The alternatives are genital feminization surgery or no intervention at all, but both carry a risk of sexual, psychological, and reproductive adverse outcomes [1,63]; an appropriate prenatal therapy would therefore lower this risk, especially in the more severe 21-OHD-null genotype group, which show worse genital surgery and psychological outcomes [60]. The inappropriate treatment of most fetuses, with their potential exposure to the following risks, must be weighed against the benefits of the minority of appropriately treated female fetuses carrying biallelic classical CAH mutations.

Animal studies have suggested potential risks of teratogenicity and alterations in brain structure and cognitive development, behavior, metabolic profile, and HPA axis; however, rodents are not a solid model in this case because of the different glucocorticoid sensitivity. Studies including non-rodent animals have focused on late pregnancy exposure to high doses of glucocorticoids for preterm births [60]. Given the hypothesized different outcomes of early vs. late exposure to glucocorticoids during pregnancy [64,65], these results are not applicable to evaluate prenatal treatment in CAH [60]. We will therefore focus on studies involving humans.

Regarding perinatal clinical outcomes, some evidence suggests that there may be an increased risk of cleft palate and other median-line alterations in patients prenatally treated with dexamethasone [16,66]. Some authors have reported an increased risk for normal to low birth weight and for cerebral palsy, albeit non-statistically significant [67]. Grunt et al. reported two cases of acute encephalopathy, one of which with permanent sequelae [68].

As for the long-term risks of prenatal therapy, they need to be further analyzed [16,60]. Several authors, mostly based in Sweden, where appropriate follow-up registries have been implemented, have reported alterations in children and adults prenatally treated with dexamethasone, mainly regarding cognitive and behavioral functions, especially in females [60,69]: increased social anxiety [70] and reduced sociability [71], impaired verbal intelligence and working memory [70,72,73], reduced cognitive abilities [74], and effects on gender role behavior [75]. One paper reported that prenatal therapy reduced cognitive abilities in non-CAH patients but improved them in CAH patients [76].

Conversely, no general differences were reported by other studies [71,77], and specifically no increase in anxiety, better sociability, and no differences in behavioral problems [76,78,79,80] and cognitive functions [70,77]. The same cohorts with reported cognitive alterations in children showed no such differences at an older age, suggesting the possibility of improvement/regression of these observations over time [81,82].

These concerns regarding mental and cognitive functions are supported by studies reporting different brain morphology in dexamethasone-exposed non-CAH fetuses [83] and by the adverse cognitive and behavioral effects observed for betamethasone, even if in different clinical settings [60,84,85].

As for metabolic and cardiovascular health, there have also been reports of a worse insulin-secreting capacity (based on insulin level evaluation) and lipid profile [86,87], with unknown long-term effects on metabolic and cardiovascular risk [1]. In theory, dexamethasone may be associated with altered renal, pulmonary, and pancreatic development, with a subsequent potential increased risk of hypertension, metabolic alterations, and allergic disorders, but none of these adverse outcomes have been clearly demonstrated [16]. A recent study showed no altered blood pressure profile in prenatally treated patients, including adults [88].

A meta-analysis of eight observational studies has not corroborated the reported findings on brain function nor metabolic profile [89]. Moreover, some studies with larger cohorts have strongly advocated prenatal therapy to be safe and effective [90].

There is a strong need for prospective research on short- and especially long-term effects of prenatal dexamethasone treatment [16,60]. Most of the cited works on this issue have been published by a Swedish research group, carrying out extensive research on these long-term effects [50]; however, the studied population is often of a small size. Follow-up registries must be implemented in other centers and multicentric studies must be carried out, as internationally advocated. Moreover, research on the hypothalamus–pituitary–adrenal axis function and metabolic and cardiovascular health outcomes should be implemented [60].

As for maternal safety, the risk of body weight increase, appearance of cushingoid features, edema, sleep, and mood disturbances should be taken into account. No increased risk of hypertension, gestational diabetes mellitus, or miscarriages has been observed in 21-OHD pregnancies [27,49,63,91,92,93].

Given the uncertainty regarding prenatal therapy fetal safety, some authors strongly oppose it [63,94], with national and international guidelines recommending it to be carried out only as experimental therapy in referral centers with appropriate informed consent and long-term follow-up registries [16,95,96]. Nowotny et al. performed a survey of 36 centers from 14 European countries to evaluate current practice of prenatal treatment for 21-OHD: 13 of these centers carry out prenatal therapy, with great variability in terms of prenatal diagnostics and therapy starting points, dose fractioning, and the absence of follow-up registries in more than half of the responding centers [50]. The authors underline how European cooperation may increase scientific data, potentially leading to more conclusive results.

It is of note that most of the above cited follow-up studies and reports include fetuses treated with dexamethasone at 20 μg/kg/day. As already mentioned, a reduced dose of 7.5 μg/kg/day has recently been suggested to lower the adverse effects associated with dexamethasone, but data are limited [61]. Moreover, new early prenatal diagnostic tests (already available yet not widespread) may reduce the number of inappropriately treated fetuses (i.e., cell-free fetal DNA testing; see Section 3.3). From this perspective, the risk/benefit ratio of prenatal therapy may improve.

The following paragraphs will present all possible scenarios based on the biological parents’ genotype.

#### 3.4.3. CAH-Affected Father and Mother

If both parents have classical CAH, there is a 100% chance of a classical-CAH-affected fetus (50% female; 50% male). In this case, only sex determination is needed to guide the decision of considering prenatal therapy because all females would be appropriately treated and all males should be excluded. If early fetal sex testing is available, waiting for its results is recommended. If early testing is not feasible, dexamethasone should be considered; if started, karyotype analysis from chorionic villus sampling should be performed as soon as possible, and in the case of a male fetus, switching back to hydrocortisone should not be delayed [90].

Conversely, if one of the two parents is affected by classical CAH and the other by non-classical CAH, the risk of having a child with classical CAH depends on their specific genotype: non-classical CAH patients may be compound heterozygous carriers of one classical-CAH mutation in 2/3 cases [2], bringing the risk of having a child with classical CAH to 50% (25% females); the remaining 50% of fetuses would be affected by compound heterozygous non-classical CAH. In this case, decisions on prenatal therapy should rely on available prenatal diagnostic tests:

- If early (i.e., results available by the eighth gestational week maximum) free fetal DNA testing is available, the decision of whether to start prenatal therapy can be made after fetal genetic testing. It is recommended to switch the patient’s therapy to hydrocortisone (if not already the chosen drug) to avoid inappropriate glucocorticoid delivery to the embryo/fetus until a prenatal diagnosis can be made on fetal DNA. In the case of a male fetus or a compound heterozygous non-classical CAH female fetus, hydrocortisone may be continued until delivery. Conversely, if a female fetus carries a biallelic classical CAH mutation, prenatal therapy should be considered.
- If free fetal DNA testing is available for fetal sex determination only, the same recommendation of switching to hydrocortisone applies. In the case of a male fetus, continuing with hydrocortisone is recommended; in the case of a female fetus, there is a 50% chance of biallelic mutation so that prenatal therapy may be considered.
- If no free fetal DNA testing is available, a prenatal diagnosis of biallelic CAH mutations could only be made after the 10th gestational week. In this case, a prenatal glucocorticoid treatment with dexamethasone before fetal genetic testing should be considered [49]; if karyotype analysis shows a male fetus, such therapy should be interrupted, with careful perinatal care to avoid acute adrenal insufficiency in the case of classical CAH [49]. The 75% risk of exposing a non-female and/or non-classical-CAH fetus to steroid excess must be taken into account [16].

#### 3.4.4. Classical-CAH Mother + Heterozygous Father

In these scenarios, the pregnant patient will already be under replacement therapy with glucocorticoids (i.e., hydrocortisone, prednisolone, or dexamethasone). Two scenarios may present:

1. If the biological father carries a non-classical-CAH heterozygous mutation, the fetus will have a 50% chance of inheriting the non-mutated allele from the father and be a healthy carrier, and a 50% chance of being a compound heterozygous of classical + non-classical CAH mutations, clinically translating in non-classical CAH. In both cases, no prenatal therapy would be useful and avoiding dexamethasone for the pregnant patient is mandatory.
2. If the father carries a classical-CAH heterozygous mutation, the risk of passing classical CAH to the child is 50%. Prenatal therapy may be considered, with prenatal diagnostic considerations as in Section 3.4.3.

#### 3.4.5. Classical-CAH Father + Heterozygous Mother

In this scenario, the fetal genetic risk evaluation is the same as in Section 3.4.4. However, being the mother a healthy carrier, prenatal therapy would carry more risks of glucocorticoid excess to her. Appropriate counselling and careful risk/benefit evaluation is crucial in this setting.

#### 3.4.6. Non-Classical-CAH-Affected Father and Mother

In this scenario, the fetal genetic risk of having classical CAH depends on the parents’ genotype. If both parents carry one classical-CAH allele as compound heterozygosity, the chance of classical CAH for the fetus is 25%. This would mean that if prenatal therapy is chosen, one in eight fetuses would be appropriately treated biallelic-mutated females, with seven inappropriately exposed to glucocorticoid excess. In this scenario, prenatal diagnosis appears to be especially important.

#### 3.4.7. Non-Classical-CAH-Affected Parent and Classical-CAH-Carrier Parent

In this case, if the parent with non-classical CAH carries one classical-CAH allele as compound heterozygosity, the chance of classical CAH for the fetus is 25%. The same considerations presented in Section 3.4.6 apply.

#### 3.4.8. Classical-CAH-Carrier Father and Mother

In this case, the fetal genetic risk of classical CAH is 25%. The same considerations presented in Section 3.4.6 apply.

#### 3.4.9. Unknown-Status Parent and CAH-Affected/-Carrier Parent

If one parent has not been genotyped, the possibility of them being a healthy carrier must be considered. The estimated prevalence of carrier status in the general population is around 1:60 but varies based on ethnic background (i.e., up to 1:3 in Ashkenazi Jews, but mostly varying between 1:55 and 70 among the Caucasian and non-Caucasian white population; not enough literature data was found for other ethnic groups) [2]. If the affected parent has classical CAH, the chance of having a child with classical CAH is therefore 1 in 120. Conversely, if the affected parent has non-classical CAH, it depends on their genotype and specific genetic counselling is advised. Carrying one classical-CAH mutation as compound heterozygosity (or as a healthy carrier) brings the risk to 1 in 240. If the non-classical CAH parent-specific genotype is not known, given the 2/3 risk of being a compound heterozygous carrier of one classical-CAH mutation, the chance of having a child affected by classical CAH is 1 in 360. In these scenarios, the risk/benefit ratio appears too high to recommend prenatal dexamethasone therapy unless early prenatal diagnosis is available.

#### 3.4.10. Other Scenarios

The remaining scenarios are: one wild-type parent and one CAH-affected parent; one parent carrying a non-classical CAH heterozygous mutation and the other carrying a classical-CAH heterozygous mutation; one parent affected by non-classical CAH and the other carrying a non-classical CAH heterozygous mutation; and both parents carrying non-classical CAH heterozygous mutations.

All these scenarios bring no risk of classical CAH for the fetus (except for the exceptionally rare cases of a de novo mutation [97]), with subsequently no risk of virilized genitalia nor adrenal crisis. There would be no benefit from prenatal treatment. Children born in this situation carry a variable risk of inheriting one mutated allele and need appropriately timed fertility and reproduction education and counselling [58].

If the biological mother is affected by CAH as in some of these scenarios, and is being treated with glucocorticoids, it is recommended that the patient continues her therapy with steroids that do not have a fetal effect during her pregnancy, i.e., switch from dexamethasone to hydrocortisone or prednisone/prednisolone/methylprednisolone, if not already used. It is advisable to switch to such therapy in advance, in patients expressing the desire for pregnancy, to avoid unvoluntary early fetal exposure. Appropriate clinical and laboratory monitoring is needed to titrate glucocorticoid and/or mineralocorticoid therapy during pregnancy [6].

## 4. Conclusions

We reviewed current research on pregnancy management and prenatal therapy in patients affected by CAH, outlining the different scenarios based on clinical and genetic features and summarizing present treatment strategies. As advocated by national and international guidelines, it is advised to start fetal prenatal treatment with dexamethasone, if deemed necessary, only in referral centers after accurate genotyping of the biological parents. The benefit/risk ratio of prenatal therapy varies according to the parents’ genotype. The importance of the implementation of long-term follow-up registries must be stressed to clarify the safety of prenatal therapy, establish clear and evidence-based treatment protocols (e.g., dosage, fractioning, starting point), and finally define whether prenatal therapy can and should be implemented in current clinical practice.

## Figures and Tables

**Table 1:** Genetic, clinical, and laboratory features of CAH.

Enzyme	21-OH		11β-OH	3βHSD2	17OH	StAR	P450scc	POR
	Classical	Non-classical						
**Gene**	*CYP21A2*		*CYP11B1*	*HSD3B2*	*CYP17A1*	*StAR*	*CYP11A1*	*POR*
**Incidence**	1:10,000–1:15,000	1:27–1:300 ^a^	1:100,000–1:200,000	<1:1,000,000	1:50,000	<1:250,000	anecdotal	anecdotal
**Congenital onset**	Yes	No	Yes	Yes	Variable ^b^	Yes	Yes	Variable
Hormonal activity *								
- Glucocorticoids	↓	=/↓	↓	↓	=	↓	↓	Variable
- Mineralocorticoids	↓/=	=/↓	↑	↓	↑	↓	↓	Variable
- Androgens	↑	↑	↑	↑	↓	↓	↓	Variable
**Clinical features**								
- Hypertension	No	No	Yes	No	Yes	No	No	Variable
- Genitalia at birth ^c^	V	N	V	V/H	F/H ^d^	F/H ^e^	F/H ^e^	Variable
- Adrenal crisis	Variable	Rare	No	Yes	No	Yes	Yes	Variable
**Biochemical hallmarks ^f^**	↑ 17-OH-P		↓ K^+^, ↑ DOC	↓ Na^+^, ↑ K^+^, 17-OH-pregnenolone, DHEA	↓ K^+^, PRA; ↑ DOC, LH, FSH, P	↓ basal and stimulated steroid hormones; ↑ PRA, LH, FSH		Variable

DOC: deoxycorticosterone; PRA: plasma renin Activity; 17-OH-pregnenolone: 17-hydroxypregnenolone; P: progesterone. * ↑: increased; =: normal; ↓: decreased. ^a^ Prevalence has been used instead of incidence. ^b^ 46,XX patients affected by 17-OHD are usually diagnosed at puberty because of primary amenorrhea, hypertension, and hypogonadism. ^c^ V = virilized, N = normal, H = hypovirilized, F = feminized. ^d^ 46,XY: ambiguous or feminized genitalia at birth; 46,XX: normal genitalia at birth but a lack of development of secondary sexual characteristics because of hypergonadotropic hypogonadism. ^e^ 46,XY: ambiguous or feminized genitalia at birth; 46,XX: normal genitalia at birth and normal puberty but anovulation due to low estrogen. ^f^ Other than elevated ACTH and normal to low cortisol levels.

## Data Availability

Not applicable.
